# Research on the epigenetic alterations in *Atractylodes macrocephala* and the synergistic regulatory mechanism of “soil environment-rhizosphere microbes” on its secondary metabolism

**DOI:** 10.3389/fpls.2026.1951327

**Published:** 2026-10-01

**Authors:** Yulong Li, Jian You, WenJing Zhang, Wei Zhao, Ming Xing, Jiangnan Li, Cui Ma, Wubuli Alimu, Yueming Zhao, Yujiao Zhang, Xia Chen

**Affiliations:** 1 National and Local United Engineering Laboratory for Chinese Herbal Medicine Breeding and Cultivation, School of Life Science, Jilin University, Changchun, Jilin, China; 2 The first hospital of Jilin University, Jilin University, Changchun, Jilin, China; 3 Akesu Prefecture Uyghur Medical Hospital, Akesu Prefecture, Xinjiang, China; 4 Changbaishan Nature Conservation Management Center & Changbaishan Academy of Science, Yanbian, Jilin, China; 5 Yanbian Specialties Research Institute, Yanbian, Jilin, China

**Keywords:** epigenetic, rhizosphere soil microorganism, secondary metabolism of Atractylodes macrocephala, soil enzymatic activities, soil physicochemical properties

## Abstract

**Introduction:**

*Atractylodes macrocephala*, a traditional Chinese medicinal herb, has a long history of cultivation and strong market demand in China. However, existing research has primarily focused on its germplasm resources and medicinal properties, while studies on the interaction mechanisms between secondary metabolism and rhizosphere soil environmental factors remain insufficient.

**Methods:**

This study investigated four *Atractylodes macrocephala* of quality grades, systematically compared their secondary metabolic pathways and rhizosphere soil environmental factors based on epigenetic variation assessments, and explored the intrinsic relationships between them using multidimensional statistical methods.

**Results:**

The results revealed that cultivated *Atractylodes macrocephala* modulates DNA methylation patterns to adapt to environmental changes, exhibiting rich epigenetic diversity. Additionally, significant differences are observed in the secondary metabolic pathways of phenylpropanoids, isoflavonoids, and flavonoids. Soil nitrogen nutrition and pH value were identified as key environmental driving forces, with efficient nitrogen transformation and weakly acidic conditions being more favorable for *Atractylodes macrocephala* cultivation. Rhizosphere fungi serve as key microbial regulators, influencing secondary metabolism through energy pathways (b-oxidation, glycolysis, glyoxylate cycle), cofactor production (ubiquinone, heme), and amino acid and lipid metabolism (nutrient supply, release of phosphatidic acid and ROS).

**Discussion:**

In contrast, bacteria mainly support soil iron nutrition, pollutant degradation, and pathogen infectivity modulation via virulence actor release. In conclusion, the secondary metabolic processes in *Atractylodes macrocephala* are synergistically regulated by an "soil environment-rhizosphere microbiota" system. These findings provide a theoretical basis for the standardized cultivation and resource development of *Atractylodes macrocephala*.

## Introduction

*Atractylodes macrocephala* Koidz., a perennial medicinal plant belonging to the genus Atractylodes of the Compositae family, is utilized in its dried rhizome form for therapeutic purposes (National Pharmacopoeia Commission, 2020). Modern pharmacological research has identified sesquiterpene lactones (such as atractylenolide (Atr) I, II, and III) and polysaccharides as the principal bioactive constituents of *Atractylodes macrocephala* (Luan et al., 2023). According to the Chinese Pharmacopoeia, this herb is traditionally recognized for its therapeutic effects in tonifying Qi and strengthening the spleen, drying dampness and promoting diuresis, as well as calming the fetus (National Pharmacopoeia Commission, 2020). As a popular bulk medicinal material, *Atractylodes macrocephala* has consistently maintained significant market demand. However, due to overexploitation of wild resources and habitat destruction, the commercial market is predominantly supplied by cultivated *Atractylodes macrocephala* (Chen et al., 2022). Consequently, research aimed at preserving the economic and medicinal value of cultivated *Atractylodes macrocephala* has become a focal point in the current field.

Modern pharmacological research primarily focuses on the active constituents of *Atractylodes macrocephala* and their mechanisms of action. Studies have demonstrated that Atr (I, II, III) exert significant inhibitory effects on various tumor cells including hepatic and renal carcinoma cells, while polysaccharides derived from *Atractylodes macrocephala* exhibit immunomodulatory activities and gastrointestinal protective effects (Zeng, 2024; Tian, 2026; Wang, 2025; He et al., 2023). Additionally, clinical efficacy of herbal formulas featuring *Atractylodes macrocephala* as a principal component, such as Shenling Baizhu San, has been reported in the treatment of chronic urticaria with spleen deficiency and dampness retention patterns (Song et al., 2015). As the industrial scale of cultivated *Atractylodes macrocephala* expands, its quality evaluation and germplasm resource conservation have become research focal points. Current research predominantly concentrates on comparing the variations of principal constituents among different geographical origins, as well as between wild and cultivated populations (Zou et al., 2026; Zhou et al., 2000; Dong et al., 2025), or assessing population survival potential and risk resistance capabilities based on genetic diversity and population genetic structure evaluations (Ma et al., 2009; Zheng, 2013). Nevertheless, studies investigating the interaction mechanisms between rhizosphere soil environmental factors and secondary metabolic pathways in cultivated *Atractylodes macrocephala* remain scarce.

The rhizosphere soil not only serves as a critical interface for plant nutrient acquisition but also acts as a pivotal driving force in the quality formation of medicinal plants (Liu F et al., 2023). Firstly, soil microorganisms, as core components of ecosystems, not only directly regulate the growth, development, and stress resistance of medicinal plants but also significantly influence the accumulation of active ingredients (Gouda et al., 2018; Shi et al., 2017; Wang et al., 2022). Their metabolite-derived soil enzymes drive nutrient transformation processes, modulate soil physicochemical properties (such as pH value and water content), and promote the efficient absorption of nutritional elements by medicinal plants (Chen et al., 2020). As important indicators of soil fertility, physicochemical properties directly constrain plant growth and have been demonstrated to significantly affect the accumulation of active components in medicinal plants (Brady and Weil, 2002; Yuan et al., 2020). Consequently, soil environmental factors profoundly influence the physiological processes of medicinal plants. Secondary metabolism, as the primary defense response mechanism in plants, its pathways are necessarily regulated by the rhizosphere environment, while metabolites can also influence the rhizosphere microecology through root exudation (Liu C et al., 2023; Zhao et al., 2023). Given that secondary metabolites directly determine medicinal value, investigating the interaction mechanisms between secondary metabolic pathways of *Atractylodes macrocephala* and rhizosphere soil environmental factors is particularly crucial.

This study, four *Atractylodes macrocephala* of quality grades, each with a different cultivation age, were selected from a medicinal plant farm in Pingjiang County, Yueyang City, Hunan Province. These varieties represent different levels of quality in cultivated *Atractylodes macrocephala*. Using ultra high-performance liquid chromatography-mass spectrometry (UHPLC-MS), we conducted comprehensive analyses of significantly different secondary metabolic pathways to investigate the primary regulatory mechanisms underlying quality variations in cultivated *Atractylodes macrocephala* across different age. Additionally, we compared the specific differences in rhizosphere soil environments (including physicochemical properties, enzyme activities, and microbial communities) among the four *Atractylodes macrocephala* of quality grades. Through a combination of mantel test, spearman’s correlation analysis, and redundancy analysis (RDA), we examined the correlations between secondary metabolic pathways and rhizosphere soil environments, thereby elucidating their interaction mechanisms. Methylation-sensitive amplified polymorphism (MSAP) markers is employed for epigenetic assessment of epigenetic structure and diversity, providing insights into the vitality and variation characteristics of germplasm resources. The present study aims to identify key factors influencing secondary metabolic pathways in *Atractylodes macrocephala*, providing theoretical foundations for standardized cultivation, development, and utilization of this important medicinal plant.

## Materials and methods

### Collection and identification of *Atractylodes macrocephala* samples with different qualities

Test samples of *Atractylodes macrocephala* were collected in November 2023 from the medicinal plant cultivation base located in Pingjiang County, Yueyang City, Hunan Province, China (N28°79′, E114°04′). This period represents the optimal harvest time for the roots of *Atractylodes macrocephala*. Four *Atractylodes macrocephala* of quality grades cultivated for varying age—1 year (B1), 2 years (B2), 3 years (B3), and 4 years (B4)—were selected as experimental materials (**Supplementary Figure S1**). These four quality grades exhibited significant differences in their major active pharmaceutical ingredients. For each cultivation duration, six plants were randomly selected. Their dried rhizomes were washed, naturally air-dried, and subsequently stored at 4 °C for further analysis. All samples were identified as *Atractylodes macrocephala* by Professor Guan Shuwen from the College of Life Sciences, Jilin University.

### Extraction and detection of secondary metabolites from *Atractylodes macrocephala*

#### Extraction process

Accurately weigh the appropriate amount of sample into a 2 mL centrifuge tube, add 600 µL of methanol (containing 2-amino-3-(2-chloro-phenyl)-propionic acid at 4 µg/mL), and vortex for 30 s. Add steel balls, then place the tube in a tissue grinder for 60 s at 55 Hz. Subject the mixture to room temperature ultrasound for 15 min, followed by centrifuged at 12,000 × g for 10 min at 4 °C. Filter the supernatant through a 0.22 µm membrane and transfer it into an autosampler vial for LC-MS analysis (Vasilev et al., 2016).

#### Mobile phase conditions

UHPLC analysis was performed on a Vanquish System (Thermo Fisher Scientific, USA) using an ACQUITY UPLC ^®^ HSS T3 (2.1×100mm, 1.8 µm) column (Waters, Milford, MA, USA) maintained at 40 °C. The flow rate and injection volume were set at 0.3 mL/min and 2 µL, respectively. For LC-HESI (+)-MS analysis, the mobile phases were (B2) 0.1% formic acid in acetonitrile and (A2) 0.1% formic acid in water. The gradient was: 0–1 min, 8% B2; 1–8 min, 8-98% B2; 8–10 min, 98% B2; 10-10.1 min, 98-8% B2; 10.1–12 min, 8% B2. For LC-ESI (-)-MS analysis, the mobile phases were (B3) acetonitrile and (A3) ammonium formate (5mM). The gradient was: 0–1 min, 8% B3; 1–8 min, 8-98% B3; 8–10 min, 98% B3; 10-10.1 min, 98-8% B3; 10.1–12 min, 8% B3 (Want et al., 2013).

#### Mass spectrometry conditions

Mass spectrometric detection of metabolites was performed on a Q Exactive HF-X (Thermo Fisher Scientific, USA) with an ESI ion source. Simultaneous MS1 and MS/MS (Full MS-ddMS2 mode, data-dependent MS/MS) acquisition was used. The parameters were as follows: sheath gas pressure, 40 arb; aux gas flow, 10 arb; spray voltage, 3.50 kV and -2.50 kV for HESI (+) and HESI (-), respectively; capillary temperature, 325 °C; MS1 range, m/z 100-1000; MS1 resolving power, 60000 FWHM; number of data dependant scans per cycle, 4; MS/MS resolving power, 15000 FWHM; normalized collision energy, 30%; dynamic exclusion time, automatic (Zelena et al., 2009).

#### Main medicinal components

This study employed the external standard method to determine the contents of major active pharmaceutical ingredients using reference standards of Atr I, II, and III (HPLC grade, purity ≥ 98%, Chem Faces) and glucose (chromatographic grade, Shanghai Ronson). The calibration curves were established as follows: Atr I (y = 537.08x + 21.452, R² = 0.999), Atr II (y = 2411.3x - 10.934, R² = 0.9997), Atr III (y = 2807.1x - 9.4128, R² = 0.9996), and atractylodes polysaccharide (y = 0.008x + 0.0032, R² = 0.9973). Notably, the atractylodes polysaccharide was determined using the anthrone-sulfuric acid method. The quantified contents were finally presented in the form of box plots.

#### QC and QA measures in the detection process of secondary metabolites

To ensure reliable and high-quality UHPLC-MS detection data, implementation of Quality Control (QC) and Quality Assurance (QA) procedures is essential throughout the analytical process (Dunn et al., 2011). QC involves insertion of identical QC samples into the sample queue to monitor variability during sample extraction and analytical runs, where smaller variability indicates higher methodological stability and superior data quality. Features were removed from the dataset based on their reproducibility in QC samples, typically in biomarker discovery applications, where the relative standard deviation (RSD) of potential biomarker features in QC samples must not exceed 30%; any features failing to meet this criterion are to be excluded from further analysis.

#### Identification and analysis of differential expression levels of secondary metabolites

Based on the molecular weight, retention time, and MS/MS spectra of features, secondary metabolites from *Atractylodes macrocephala* were identified through database matching against TCM spectral libraries. The Euclidean distances of relative metabolite abundances were calculated to perform hierarchical clustering analysis, constructing heatmaps and dendrograms to evaluate metabolic profile similarities and expression patterns among different quality *Atractylodes macrocephala* samples. Subsequently, partial least squares-discriminant analysis (PLS-DA) was employed to establish regression models, maximizing inter-group differences while minimizing intra-group differences. Combined with Variable Importance in Projection (VIP) scores and p-values, significantly differentially expressed metabolites were screened and their expression patterns visualized. Finally, pathway enrichment analysis based on the hypergeometric test integrated with topological analysis (based on betweenness centrality) was used to screen key metabolic pathways: the former assessed enrichment significance of metabolites, while the latter evaluated their structural importance within pathways. Through comprehensive analysis of impact values (reflecting relative importance of metabolites) and p-values, differentially expressed secondary metabolic pathways were ultimately determined. The Impact value was calculated as: Impact = Σ(scores of matched metabolites)/Σ(scores of all metabolites in the pathway). To confirm the PLS-DA model’s validity, we evaluated its robustness through permutation tests, observing that the predicted R2 and Q2 values were below the original values in the top right. Cross-validation also revealed R2Y and Q2 values above 0.9 (**Supplementary Figure S6c-d**). Moreover, we employed the Benjamini-Hochberg method to calculate the False Discovery Rate (FDR) for p-value adjustment.

#### Detection of epigenetic alterations in *Atractylodes macrocephala*

In this study, fresh leaf samples of *Atractylodes macrocephala* were collected in May 2023, which avoided the leafless period in autumn and winter and the high-temperature period in summer, ensuring mature leaves were selected. We focused on analyzing CG methylation patterns, known for their minimal seasonal variation. From each plot reflecting different cultivation age, we randomly selected 15 plants at a uniform growth stage for biological replicates. Approximately 3–4 healthy leaves per plant were collected, stored in silica gel-lined bags to maintain dryness, and one leaf from each bag was used for analysis. DNA extraction was performed using a plant genomic DNA extraction kit from Tiangen (Beijing). The MSAP analysis involved restriction enzymes (EcoR I, Msp I, Hpa II) from New England Biolabs (Beijing), and MSAP adapters from Sangon Biotech (Shanghai). Primer sequences are provided in **Supplementary Table S1** (Ni, 2022). The single-stranded adapters were annealed into double-stranded adapters (95°C, 5min; 65°C, 10min; 4°C, 10min) and stored at -20°C. Subsequent digestion and ligation reactions were performed simultaneously (digestion 37°C, 12h; ligation 16°C, 4h; 65°C, 10min). Pre-amplification was carried out with E0 and HM0: 94°C, 5min; 35 cycles (94°C, 30s; 56°C, 30s; 72°C, 80s); 72°C, 10min, and stored at 4°C. The pre-amplification products were diluted 50-fold and combined with 6 pairs of primers for MSAP selective amplification. The reaction used a 2× EasyTaq PCR Supermix for PAGE system (Quanshijin, Beijing) with the following conditions: 94°C, 5min; 12 cycles (94°C, 30s; 65°C (decreasing 0.7°C per cycle), 30s; 72°C, 90s); 72°C, 10min; 23 cycles (94°C, 30s; 56°C, 30s; 72°C, 90s); 72°C, 10min, and stored at 4°C. The amplified products were detected by 6% denaturing polyacrylamide gel electrophoresis: 80W, 30min; 75W, 90min. The final results are shown in **Supplementary Figure S2**.

#### Analysis of epigenetic alterations

The study identified four methylation patterns in *Atractylodes macrocephala*: Type I (digested by both Hpa II and Msp I, representing unmethylated sites), Type II (digested by Hpa II, representing hemimethylated sites), Type III (digested by Msp I, representing fully methylated sites), and Type IV (no enzyme digestion bands). These patterns, illustrated in **Supplementary Figure S2**, were used to create a binary matrix where Types II and III were coded as “1” and others as “0”. Methylation levels were calculated as follows: hemimethylation level = (II/(I + II + III)) * 100%; full methylation level = (III/(I + II + III)) * 100%; total methylation level = ((II + III)/(I + II + III)) * 100%. This matrix was then used to analyze the epigenetic diversity of various *Atractylodes macrocephala* strains using POPGENE32 software. Additionally, AMOVA and PCoA analyses were conducted with GENEALEX6.5, both with a 95% confidence interval.

#### Detection of rhizosphere soil physicochemical properties and enzyme activities

Soil samples from the rhizosphere of different *Atractylodes macrocephala* cultivars were collected concurrently. For each variety, a 5-point sampling method was used to collect rhizosphere soil, which was then combined into one composite sample. Sampling was performed in triplicate, yielding three composite samples per grades. Three composite sample were processed according to the standard Evaluating the Measurement Process protocol, falling within the significance evaluation threshold. Statistical analysis using a t-distribution yielded a 95% confidence interval (CI) = $\bar{x}$ ± 2.484s. Some samples were stored at -80 °C for subsequent microbial analysis, while others were stored at 4 °C for water content measurements. The remaining samples were air-dried, ground, passed through a 60-mesh sieve, and stored at room temperature. Soil moisture content (SMC) was calculated using a formula: [(fresh soil + box mass) –(oven-dried soil + box mass)/[(fresh soil + box mass) – (dried box mass)] * 100%. Soil pH was measured by mixing soil with water at a soil-to-water ratio of 1:5 (w/v), stirring for 1 min, allowing to stand for 30 min, and then measuring with a pH meter. Total nitrogen (TN) was measured by the Kjeldahl method, soil organic carbon (SOC) content by the potassium dichromate-concentrated sulfuric acid oxidation heating method, NH_4_^+^-N content by the 1 mol L^-^¹ KCl extraction–indophenol blue colorimetric method, NO^-3^-N content by the phenol disulfonic acid colorimetric method, soil sucrase (S-SC) activity by the 3,5-dinitrosalicylic acid colorimetric method, soil alkaline phosphatase (S-ALP) activity by the disodium phenyl phosphate colorimetric method, soil catalase (S-CAT) activity by the potassium permanganate titration method, and soil urease (S-UE) activity by the sodium phenoxide-sodium hypochlorite colorimetric method.

#### Statistical analysis of significant differences

To ensure reliable detection of key parameters like medicinal components, soil properties, and enzyme activities, we conducted three biological replicates for each sample. These replicates enhance data reliability. We first assessed data normality using the Shapiro-Wilk test, crucial for selecting appropriate statistical methods. Then, we checked variance homogeneity across groups with the Levene test. If data met both normality and homogeneity criteria, we employed ANOVA followed by Tukey’s HSD test to identify significant differences. Otherwise, we used the Kruskal-Wallis and Dunn tests, suitable for non-parametric data. To correct for multiple comparisons, we applied the Bonferroni method to adjust p-values. Beyond p-values, we also evaluated the effect size between samples, a measure of the practical significance of differences, using Cohen’s d. To refine this measure, we calculated Hedges’ g, a bias-corrected version of Cohen’s d. Finally, we integrated these findings and visualized them using R, providing a comprehensive assessment of sample differences.

#### Detection of the composition of rhizosphere soil microbial communities

Genomic DNA was extracted from rhizosphere soil samples, stored at -80 °C, using the OMEGA Soil DNA Kit. The extracted DNA was then sent to Personalbio Company for bacterial 16S rRNA and fungal ITS sequencing. The resulting sequencing data were processed using the DADA2 algorithm for noise reduction, assembly, and chimera removal (Callahan et al., 2016). Taxonomic analysis of the bacteria and fungi was performed using the Greengenes and UNITE databases, aided by QIIME2 (v2019) software (DeSantis et al., 2006; Koljalg et al., 2013). This analysis determined the number of taxonomic units in each sample at various taxonomic levels. QIIME2 (v2019) was also used to visualize the species composition distribution of each sample at each taxonomic level. Furthermore, QIIME2 was employed to analyze the α-diversity and β-diversity of the microbial communities, with richness assessed by the Chao1 and Observed species indices, and diversity evaluated using the Shannon and Simpson indices. The differences between samples were visualized through PCoA and hierarchical clustering trees (Whittaker, 1972). A heatmap was also constructed using the top 20 most abundant species, illustrating the structure of the microbial community in the rhizosphere soil of different quality *Atractylodes macrocephala*.

#### Predictive analysis of the functions of rhizosphere soil microbes

LEfSe (LDA Effect Size) is an analytical approach that integrates non-parametric Kruskal-Wallis and Wilcoxon rank sum tests with the effect size of Linear Discriminant Analysis (LDA). This method was utilized to assess differences in microbial composition among *Atractylodes macrocephala* of different quality grades, with PLS-DA employed to identify the top 20 significantly different microorganisms (Segata et al., 2011). Phylogenetic Investigation of Communities by Reconstruction of Unobserved States (PICRUSt2), a software tool, predicts functional abundance based on marker gene sequence data in samples. It has evolved beyond relying on the Greengene database for sequence comparison, instead utilizing a pre-built phylogenetic tree within the software for taxonomic annotation of OTUs. This innovation allows PICRUSt2 to expand its predictive capabilities to include both bacterial (16S rRNA) and fungal (ITS) functional profiling. The process involves aligning reference 16S rRNA or ITS sequences from known genomes with the target sequences to construct an evolutionary tree. Using the Castor algorithm, the software predicts the nearest related species by analyzing hidden states and gene family copy numbers in the tree. It then calculates the gene family copy numbers for each sample based on the abundance of feature sequences, without merging different functional units. Finally, gene families are mapped to the MetaCyc, KEGG, and COG databases, with MinPath used to infer the presence and abundance of metabolic pathways in each sample, thus enabling accurate functional prediction (Douglas et al., 2020). PLS-DA was employed a second time to identify the top 20 microbial metabolic pathways. Additionally, the previously described method was used to validate the PLS-DA model’s results, ensuring their reliability and accuracy(**Supplementary Figure S7b, d**).

#### Mantel test and correlation analysis

This study employed the R language dplyr package to conduct Spearman correlation analysis between soil physicochemical properties, enzyme activities, microbial metabolic pathway data, and key substances from distinct metabolic pathways of different quality *Atractylodes macrocephala*. Spearman correlation was also applied among the detected soil physicochemical properties, enzyme activities, and microbial metabolic pathways. The dplyr and linkET packages in R were utilized for mantel tests and redundancy analysis, exploring the relationships between soil properties, enzyme activities, and the significantly different metabolic pathways of *Atractylodes macrocephala*. To ensure robust results, all analyses were subjected to 9999 permutations to mitigate overfitting. Additionally, we conducted mantel tests, spearman correlation analyses, and redundancy analyses, each with 9999 permutations to account for variability. Zero variance filtering was applied to ensure robustness. P-values were then corrected using the Bonferroni method, thereby enhancing the stability and precision of the final results.

## Result

### Comparative analysis of differential secondary metabolism in *Atractylodes macrocephala* with different qualities

The secondary metabolism of medicinal plants shows remarkable adaptability to environmental conditions, with the synthesis and distribution of their secondary metabolites being influenced by habitat factors. This makes them crucial indicators for assessing the quality and development potential of TCM (Zeng, 2024). In this study, we used UHPLC-MS technology to examine the variations in secondary metabolite profiles among four *Atractylodes macrocephala* of quality grades, constructing fingerprint profiles (**Supplementary Figure S3**). Rigorous Quality Control (QC) and Quality Assurance (QA) protocols were implemented to ensure data reliability. Results showed that QC samples clustered within the 95% confidence interval, and QA analysis indicated a stable proportion of features with RSD<30% at around 65%, confirming the high stability of the data (**Figures 1a, b**; **Supplementary Figure S4**). Our analysis identified 2326 chemical components, with 1152 structures confirmed. Flavonoids were the most abundant (25%), followed by terpenoids (17.88%) (**Figures 1c, d**). Venn diagram analysis revealed a significant overlap in chemical components among the *Atractylodes macrocephala* of different quality grades (**Supplementary Figure S5**). As Atr and atractylodes polysaccharide are key bioactive components, their absolute contents in the four quality grades were determined using an external standard method. Significant differences were found in Atr I and II contents among samples (p<0.001, Hedges’ g > 0.8), but no difference in atractylodes polysaccharide content. Notably, the grades B2 had the highest Atr-II content, while the grades B3 had the highest Atr-I content (**Figures 1e–h**).

**Figure 1:**
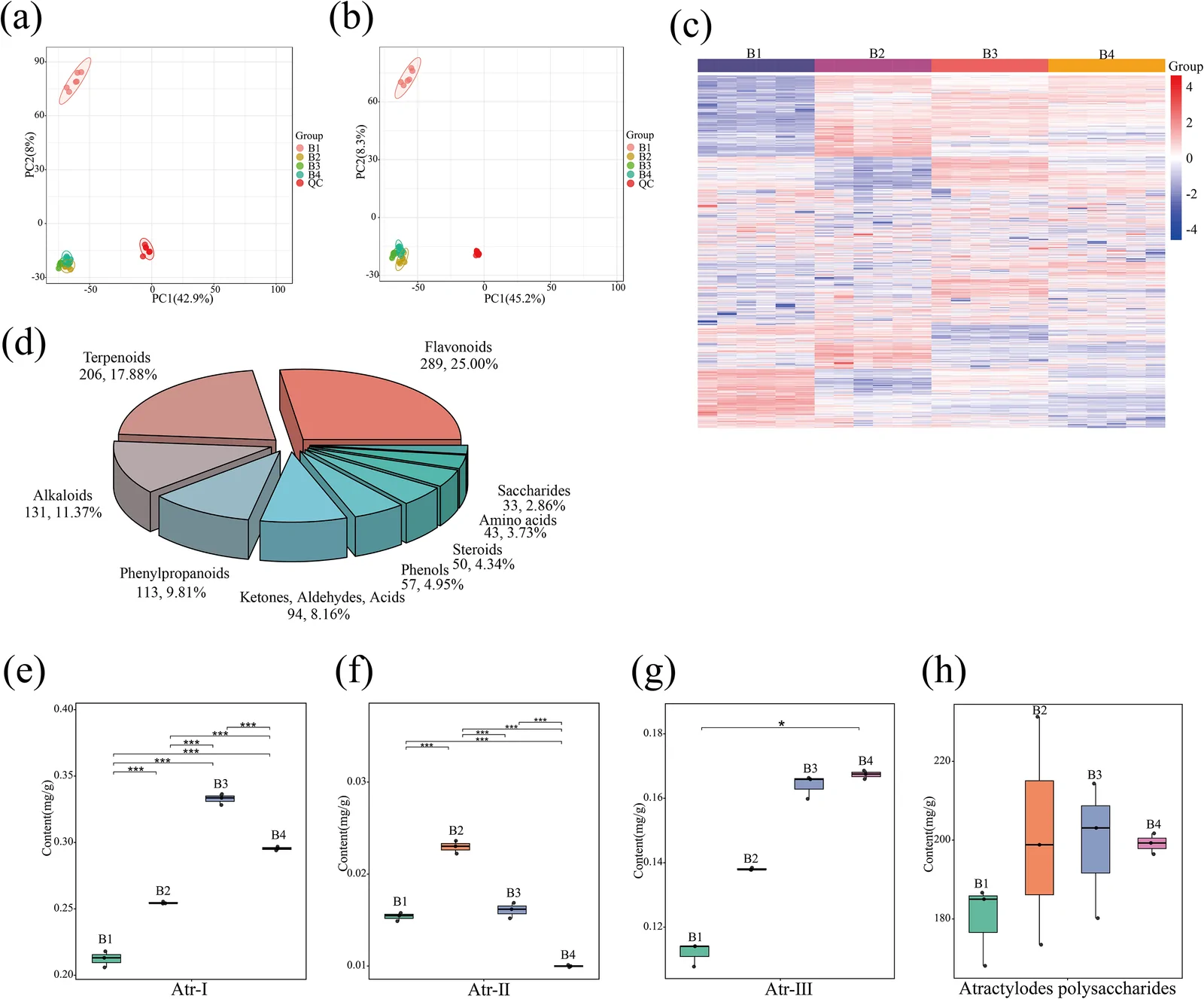
Identification of secondary metabolites in *Atractylodes macrocephala* and comparison of key medicinal component levels. **(a, b)** Analysis of QC and QA for secondary metabolites, with red dots representing QC samples. The closer grouping within the 95% confidence interval indicates better detection repeatability. **(c)** Heatmap displaying secondary metabolites, where colors represent different relative levels, with red indicating high levels and blue indicating low levels. **(d)** Categorized display of secondary metabolites. **(e–h)** Comparison of the levels of key medicinal components—Atr I, II, III, and atractylodes polysaccharides. Statistical significance is denoted by “*” = p<0.05, “**” =p<0.01, and “***” = p<0.001, indicating increasingly significant differences.

Given the differences in quality among the *Atractylodes macrocephala*, we constructed a hierarchical clustering tree based on the relative contents of chemical components to assess the variation in secondary metabolites. The results showed that the grades B3 and B4 clustered together, indicating similar secondary metabolite profiles, while the grades B1 formed a separate branch, suggesting significant differences in its secondary metabolites compared to the others (**Figure 2a**). To confirm this, we conducted principal component analysis (PCA), which similarly showed that the grades B3 and B4 were close along the PC1 axis, with the grades B1 being the most distant (**Supplementary Figures S6a, b**).

**Figure 2:**
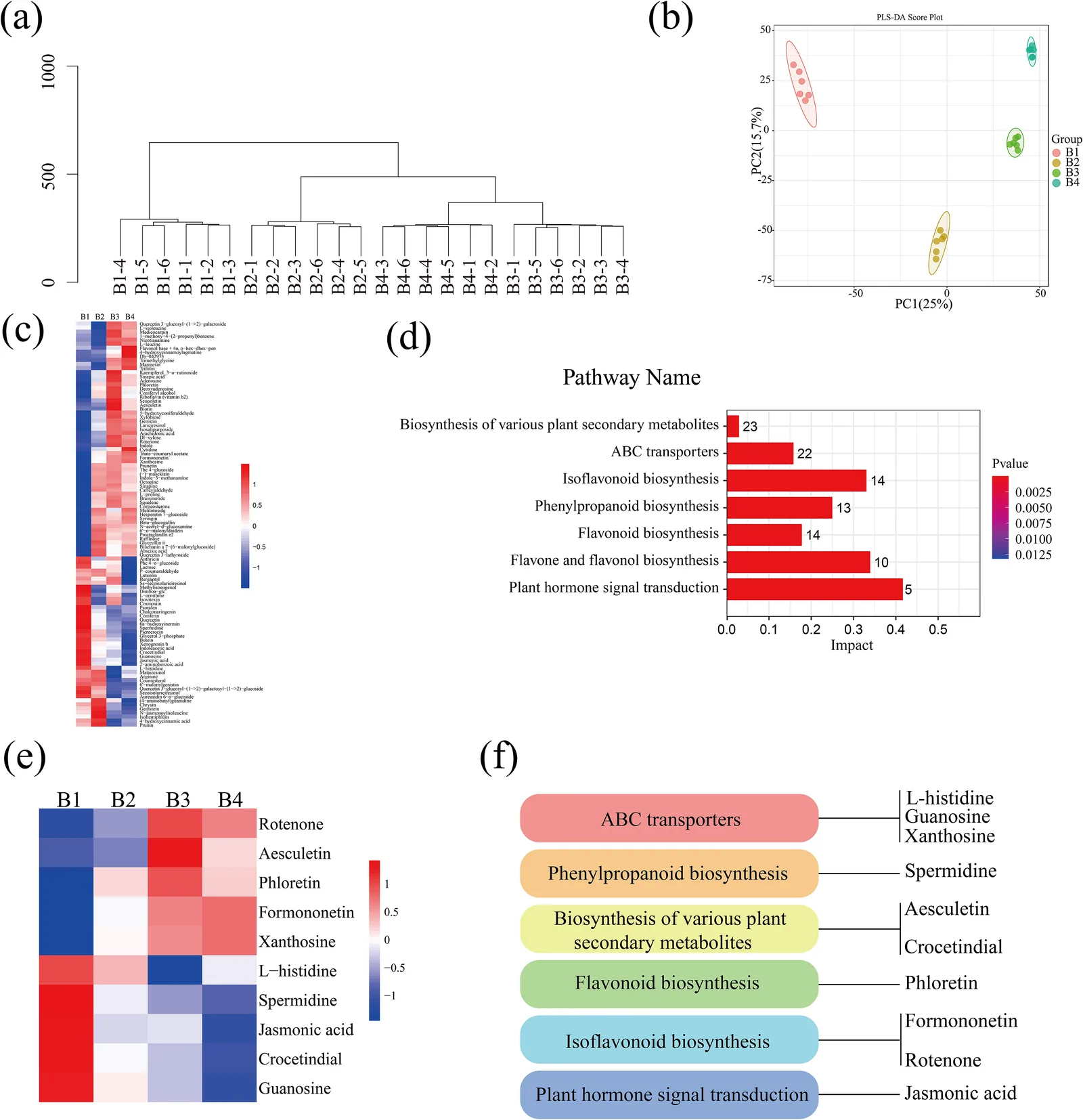
Analysis of Differential Metabolites in Atractylodes macrocephala. **(a)** Hierarchical clustering based on the Euclidean distance of the relative levels of secondary metabolites. **(b)** PCA-based ordination of Atractylodes macrocephala samples derived from the relative levels of secondary metabolites, with 95% confidence ellipses. **(c)** Heatmap displaying the differential metabolites in Atractylodes macrocephala of different quality grades, where red indicates high levels and blue indicates low levels. **(d)** Top 6 significantly differential metabolic pathways, with the X-axis representing the pathway impact value, and the color indicating the p-value, which is significant at p<0.001. **(e)** Top 10 representative substances of significantly differential secondary metabolites, selected from all differential metabolic pathways, p<0.001. **(f)** Classification of significantly differential metabolic substances according to their respective metabolic pathways.

These findings suggest that the four *Atractylodes macrocephala* of quality grades have distinct secondary metabolite profiles, with the grades B3 and B4 showing more similar patterns. The unique profile of the grades B1 may be due to its shorter cultivation time and lower secondary metabolite accumulation, while the grades B2 exhibits intermediate characteristics between the grades B3 and B4.

Partial least squares discriminant analysis (PLS-DA) is a supervised statistical method that quantifies the contribution of metabolites to sample classification through variable importance in projection (VIP) scores. We applied this model to classify four *Atractylodes macrocephala* of quality grades, with results consistent with PCA (**Figure 2b**; **Supplementary Figure S6c** Q2 = 0.992). Combining VIP values with significance testing, we identified differential metabolites, revealing that the grades B1 had the highest number of downregulated metabolites compared to other (**Figure 2c**, p < 0.001, FDR < 0.001). Further analysis using hypergeometric testing and betweenness centrality topological analysis, coupled with KEGG databaseretrieval, identified seven significantly different metabolic pathways (**Figure 2d**, p<0.05, FDR < 0.05). These pathways primarily encompassed synthetic routes from phenylpropanoid biosynthesis to *Atractylodes macrocephala* -specific secondary metabolites, providing evidence for the formation of significant grades characteristics in secondary metabolism among *Atractylodes macrocephala* of different quality grades. We selected the top ten differential metabolites for analysis, finding that their corresponding differential metabolic pathways were highly concentrated within 6 of the seven effective differential pathways identified above (**Figures 2e, f**; **Supplementary Figure S6d** Q2 = 0.938, p<0.05). This study focused specifically on these six core metabolic pathways to clarify the precise patterns of *Atractylodes macrocephala* secondary metabolic network differentiation. As illustrated in **Figure 3**, *Atractylodes macrocephala* perceives environmental stimuli through plant hormone signaling pathways, among which jasmonic acid (JA) fluctuations were most significant. These signaling pathways transmit activation signals to metabolic pathway transcription factors via ABC transporter pathways, thereby regulating phenylpropanoid biosynthesis and its specialized branches (flavonoids/isoflavonoids), ultimately affecting the production of secondary metabolites such as atractylodes polysaccharide and Atr. The synthesized secondary metabolites not only exert defensive functions (such as isoflavones as phytoalexins) or growth-regulatory effects (such as flavonoids modulating auxin transport), but also feedback-regulate environmental microbial composition through ABC transporter pathways via root exudation from *Atractylodes macrocephala*, enabling better adaptation to habitats. Compared to others, the secondary metabolic pathways of the grades B2 operated more stably, with balanced secondary metabolite production and no marked specificity. Regarding major medicinal components, the grades B2 exhibited the highest content of Atr-II, with only Atr-I significantly lower than in the grades B3(p < 0.001, Hedges’ g > 0.8). In summary, *Atractylodes macrocephala* secondary metabolic pathways exhibit clear differentiation mechanisms, and the grades B2, with its stable metabolic characteristics and superior component performance, demonstrates greater potential for medicinal material development.

**Figure 3:**
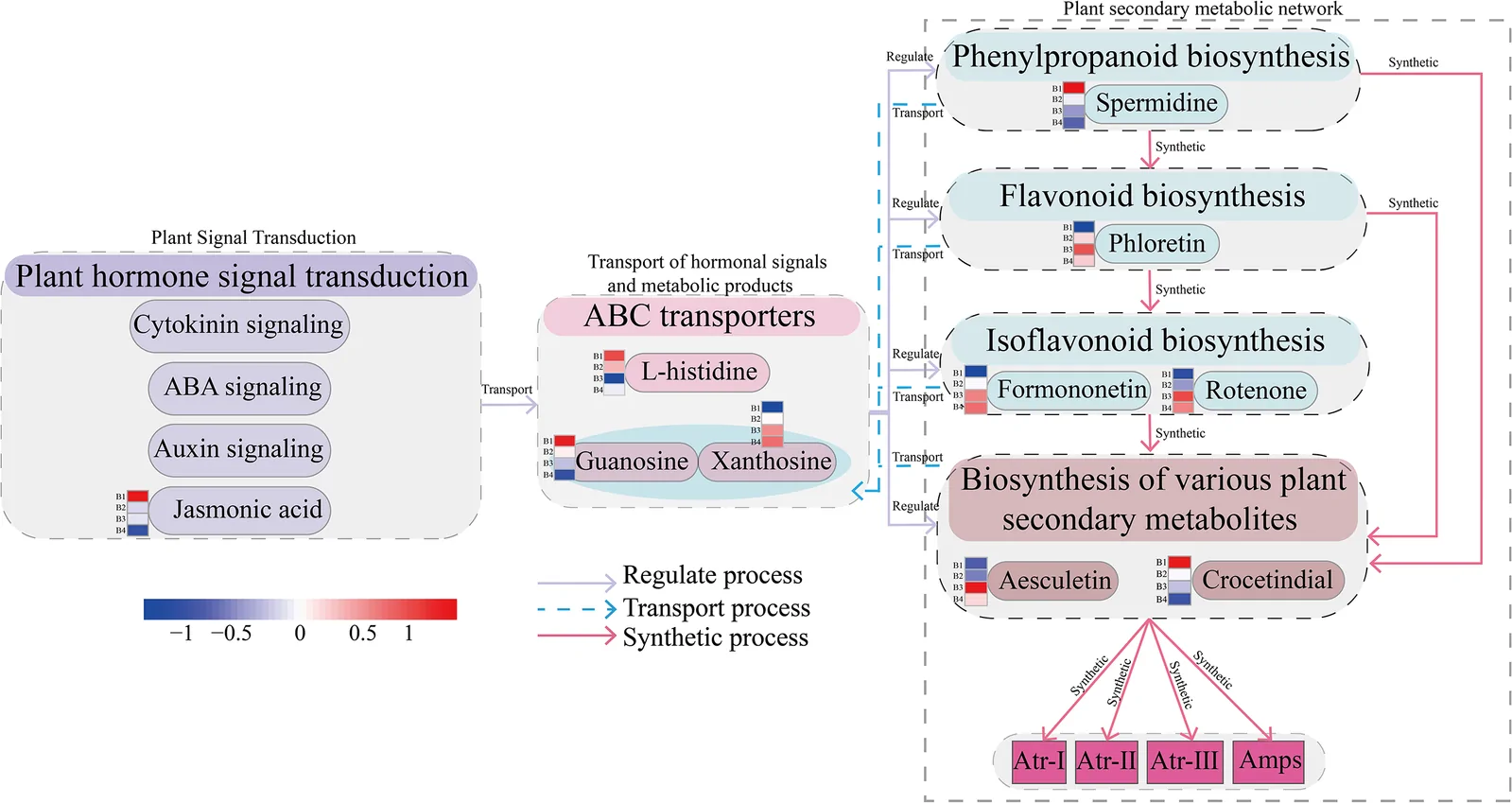
Response mechanism of key differential secondary metabolic pathways in *Atractylodes macrocephala*. This process begins with the plant hormone pathway, is transmitted via the ABC transport pathway, and is specifically regulated by the phenylpropanoid backbone secondary metabolic pathway. Additionally, the secondary metabolites of the products are feedback-regulated through the ABC transport pathway. The figure also displays the differential expression levels of representative metabolites in each pathway, with statistical significance at p<0.05 and FDR < 0.05.

### Analysis of epigenetic differentiation and diversity in *Atractylodes macrocephala* with different qualities

Epigenetic variations, such as DNA methylation, are highly sensitive to environmental changes and are closely associated with the growth, development, and metabolism of medicinal plants (Guo et al., 2021). Therefore, we employed MSAP molecular marker technology to analyze the degree of methylation variation among *Atractylodes macrocephala* of different quality grades. The results revealed that *Atractylodes macrocephala* exhibited overall high methylation levels, with total methylation rates exceeding 50% and fully methylated levels surpassing 25%, with the B3 reaching up to 29% (**Figure 4a**; **Supplementary Table S2**). Further analysis indicated that the epigenetic differentiation coefficient (Gst) among samples was 0.2038, indicating substantial epigenetic differentiation, and approximately 80% of the variation was observed within sample of different quality grades (**Table 1**). This suggests that the significant epigenetic differences observed among *Atractylodes macrocephala* of different quality grades are due to internal adjustments in DNA methylation patterns. PCoA further validated this inference: on the PCo1 and PCo2 axes, the grades B1 and B4 were clearly separated, forming distinct clusters; while the overlapping area between the grades B2 and B3 began to decrease, showing a trend toward independent differentiation (**Figure 4b**, p < 0.001). This indicates that four *Atractylodes macrocephala* of quality grades have developed unique epigenetic characteristics at the epigenetic level. In summary, *Atractylodes macrocephala* of different quality grades show unique DNA methylation patterns within a short cultivation age, actively adapting to complex environmental fluctuations.

**Figure 4:**
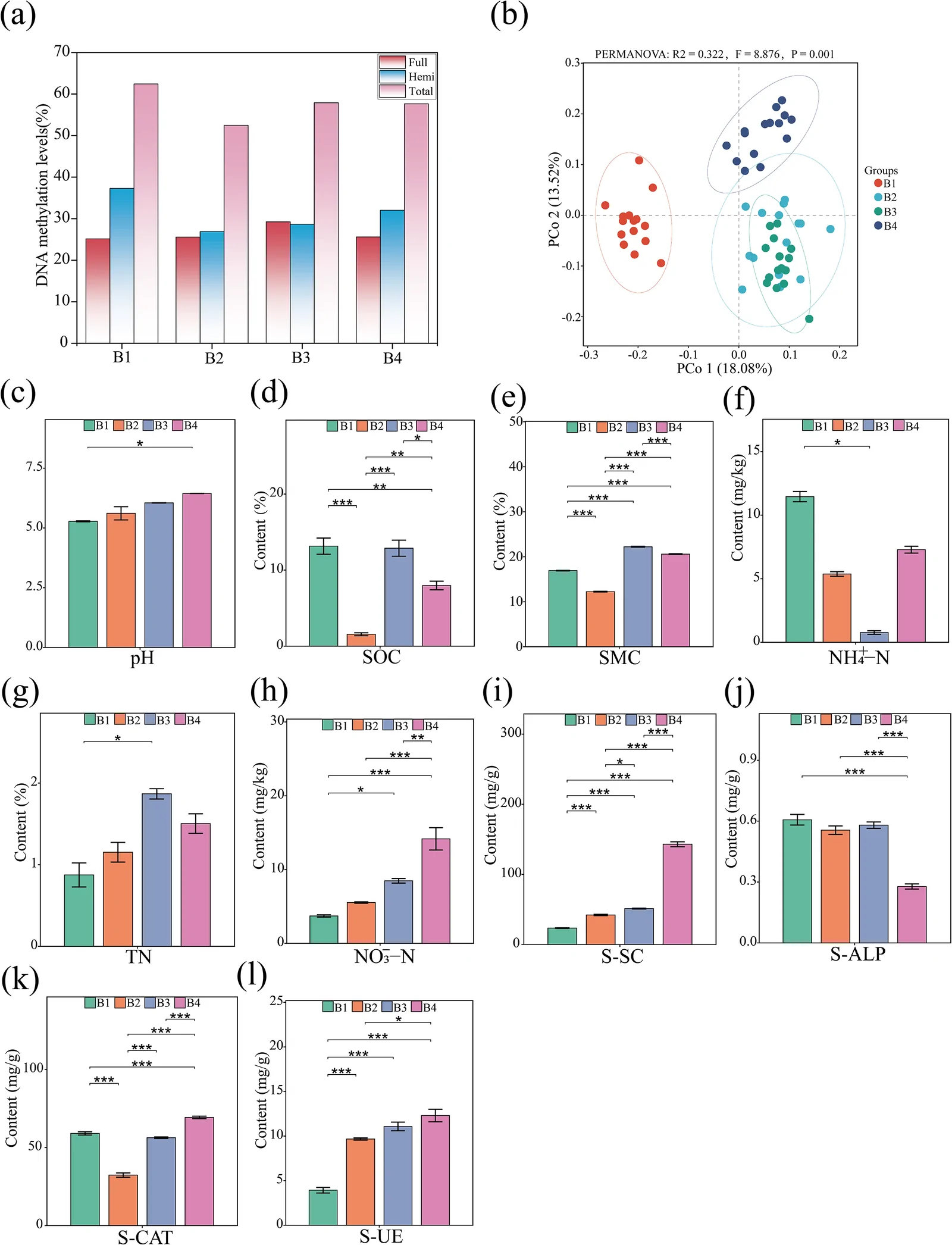
Detection of epigenetic alterations in *Atractylodes macrocephala* and analysis of differences in rhizosphere soil physicochemical properties and enzyme activities. **(a)** Presentation of methylation types in *Atractylodes macrocephala* of different quality grades. **(b)** PCoA ordination based on the extent of epigenetic alterations in *Atractylodes macrocephala*, with a 95% confidence interval. **(c–l)** Analysis of significant differences in rhizosphere soil physicochemical properties and enzyme activities among *Atractylodes macrocephala* of different quality grades, where “*” = p<0.05, “**”= p<0.01, and “***” = p<0.001, indicating increasingly significant differences.

**Table 1:** AMOVA for epigenetic variation in *Atractylodes macrocephala* of different quality grades.

Variation method	Source	df	SS	MS	Est. Var	%	*Gst*	*Nm*	*P*
Epigenetic									
	Among Pops	3	287.367	95.789	5.049	20%			
	Within Pops	56	1122.800	20.050	20.050	80%			
	Total	59	1410.167		25.099	100%	0.2038	1.9536	<0.001

Further statistical analysis of the epigenetic diversity in *Atractylodes macrocephala* revealed exceptionally high levels of epigenetic variation. At the species level, the percentage of polymorphic loci (PPL) exceeded 95%, while expected heterozygosity (He) and Shannon’s diversity index (I) surpassed 0.30 and 0.49, respectively, demonstrating high epigenetic diversity parameters (**Table 2**). At the population level, all samples exhibited similarly high epigenetic diversity indices, which suggests that the phenotypic response of *Atractylodes macrocephala* to environmental stimuli is particularly sensitive (**Table 2**). This finding strongly supports the previously proposed perspective that in the process of responding to external environmental stimuli, variations in DNA methylation patterns are the primary driver of epigenetic differentiation.

**Table 2:** Epigenetic diversity in *Atractylodes macrocephala* of different quality grades.

Variation method	Grades	*PPL*	*Na*	Ne	*He*	*I*
Epigenetic						
	B1	74.47%	1.7447	1.4200	0.2479	0.3748
	B2	80.14%	1.8014	1.4608	0.2707	0.4079
	B3	74.47%	1.7447	1.4435	0.2588	0.3880
	B4	79.43%	1.7943	1.4870	0.2843	0.4246
	Mean	77.13%	1.7713	1.4528	0.2654	0.3988
	Species	95.74%	1.9574	1.5690	0.3334	0.4990

### Analysis of differences in rhizosphere soil physicochemical properties, enzyme activities, and microbial composition in *Atractylodes macrocephala* with different qualities

Given that methylation variation, as a dominant factor affecting *Atractylodes macrocephala* secondary metabolism, is highly sensitive to external environmental changes (especially soil environmental fluctuations), we further investigated the specific differences in rhizosphere soil environment among *Atractylodes macrocephala* of different quality grades. First, analysis of rhizosphere soil physicochemical properties revealed significant differences among the four quality grades (p<0.01, Hedges’ g > 0.8). Specifically, the grades B1 exhibited the highest soil organic carbon (SOC) and ammonium nitrogen (NH_4_^+^-N) contents (max Hedges’ g = 16.3), while the grades B2 showed significantly lower SOC content compared to other populations; the grades B3 had the highest soil moisture content (SMC) and total nitrogen (TN) levels (max Hedges’ g = 54.15), whereas the grades B4 dominated in nitrate nitrogen (NO^-3^-N) content and pH value (**Figures 4c–h**). Secondly, regarding rhizosphere soil enzyme activities, we focused on measuring soil urease (S-UE), soil sucrase (S-SC), soil catalase (S-CAT), and soil alkaline phosphatase (S-ALP). Results demonstrated that these four enzyme activities also exhibited significant differences among the four populations (p<0.01, Hedges’ g > 0.8): the B4 showed the highest activities in three enzymes except ALP (max Hedges’ g = 22.24), while the B1 had the highest ALP activity (**Figures 4i–l**). These findings confirm that rhizosphere soils of *Atractylodes macrocephala* of different quality grades exhibit distinct population-specific characteristics in both physicochemical properties and enzyme activities.

Soil environmental heterogeneity often leads to differences in rhizosphere microbial communities. Therefore, we employed 16S rRNA and ITS amplicon sequencing to analyze the composition of bacterial and fungal communities in the rhizosphere soil of *Atractylodes macrocephala*. Given that bacteria exhibit more precise taxonomic annotations and richer taxonomic units at the genus level (**Figures 5d–g**), subsequent analyses primarily focused on the genus-level characteristics of bacterial communities. In terms of taxonomic composition, the relative abundance of the top 20 bacterial genera exhibited a decreasing trend from the grades B1 to B4, with the dominant genera in the grades B1 showing the highest abundance (**Figure 5c**). However, α-diversity analysis (Chao1, Simpson, Shannon, and Observed species indices) revealed that diversity levels gradually increased from the grades B1 to B4 (**Figures 5d–g**). This indicates that the *Atractylodes macrocephala* rhizosphere bacterial communities exhibit an evolutionary trend from “low diversity, dominated by few genera” to “high diversity, evenly distributed across various taxa” from the grades B1 to B4, with B1 exhibiting a highly uneven community structure. β-diversity analysis further revealed significant differentiation among samples: cluster analysis showed that the grades B3 and B4 communities exhibited similar structures and clustered together, while the grades B1 differed significantly from other populations, forming a separate cluster (**Figure 5h**). The results of PCoA analysis based on β-diversity were consistent: on the PCo1 and PCo2 axes, the grades B3 and B4 were closer in distance, validating the above conclusion (**Figure 5i**). LEfSe differential analysis identified specific bacterial groups unique to each population (LDA > 4.0) and selected the top 20 bacterial genera for presentation. The results showed that the dominant bacterial community in the grades B1 did not overlap with those of others, once again confirming the uniqueness of its community composition (**Figure 5j**; **Supplementary Figure S7a**, p < 0.05). To investigate the impact of community differences on soil ecological functions, we predicted the functional potential of bacterial communities using PICRUSt2 and selected the top 20 metabolic pathways through PLS-DA. Heatmap analysis revealed that the grades B1 exhibited the most upregulated metabolic pathways, while the grades B4 showed the most downregulated pathways (**Figures 5k, l**; **Supplementary Figure S7b** Q2 = 0.996, **Supplementary Figure S8a**, p < 0.001, FDR < 0.001), indicating significant functional differentiation in rhizosphere bacteria among *Atractylodes macrocephala* of different quality grades.

**Figure 5:**
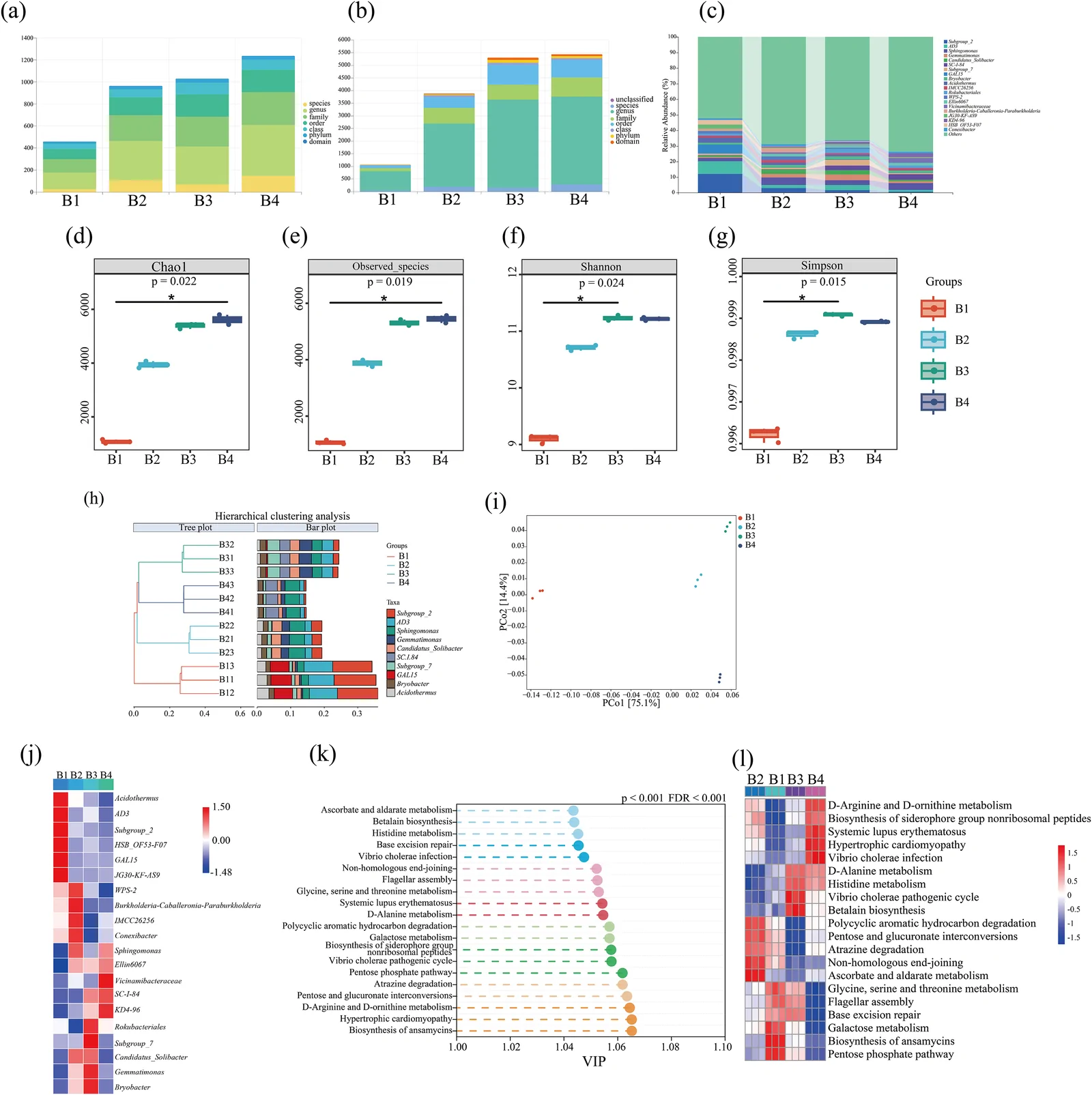
Bacterial community composition and functional analysis. **(a)** Quantitative statistics of bacterial taxonomic units. **(b)** Statistical overview of bacterial species taxonomic annotations. **(c)** Statistical analysis of bacterial community composition at the genus level. **(d–g)** Analysis of α-diversity in bacterial communities, using Chao1 and Observed species indices to evaluate richness, and Shannon and Simpson indices to evaluate diversity. **(h)** β-diversity (between-community diversity) clustering analysis of bacterial communities. **(i)** PCoA classification of bacterial community β-diversity, with a 95% confidence interval. **(j)** Heatmap depicting differential bacterial community composition at the genus level, with red indicating high abundance and blue indicating low abundance. **(k)** Prediction and top 20 selection of significantly differential metabolic pathways in bacterial communities, with statistical significance at p<0.001. **(l)** Heatmap display of the top 20 significantly differential metabolic pathways in bacterial communities, indicating high expression with red and low expression with blue.

The taxonomic classification of fungi revealed more precise species-level annotations and a richer diversity of taxonomic units at the species level (**Figures 6a, b**). Consequently, subsequent analyses primarily focused on fungal community characteristics at the species level. In terms of taxonomic composition, the top 20 abundant rhizosphere fungal species exhibited significant succession patterns among the four samples, with each sample displaying distinctive community structures. Notably, the abundance of dominant fungal genera in the grades B1 was significantly higher compared to others (**Figure 6c**). α-diversity analysis revealed that the fungal community diversity in the grades B1 was significantly lower (p<0.05), while no significant differences were observed among the grades B2, B3, and B4 (**Figures 6e–g**). This indicates that the grades B1 rhizosphere fungal community structure is simpler, characterized by “low diversity, high dominance,” with dominance by a few taxa, whereas the other populations exhibit “high diversity, relatively high dominance” characteristics, suggesting more significant variations in rhizosphere fungal community structures among *Atractylodes macrocephala* of different quality grades. β-diversity analysis demonstrated that the fungal community structures of the grade B3 and B4 were more similar, which is consistent with the bacterial community analysis results (**Figures 6h, i**). The LEfSe differential analysis identified unique dominant fungal groups (LDA>4.0), with the top 20 fungal species displayed, showing that the grade B1 possessed more dominant fungal communities, further confirming its distinctive community composition; meanwhile, the numbers of dominant communities in the grade B2, B3, and B4 were similar with minimal overlap, also reflecting high fungal community specificity (**Figure 6j**; **Supplementary Figure S7c**, p<0.05). Furthermore, PICRUSt2 prediction analysis of fungal community functional potential revealed significant differentiations in metabolic functions among samples: the grades B1 fungal community showed the highest number of upregulated metabolic pathways, while the grades B2 exhibited the fewest (**Figures 6k, l**; **Supplementary Figure S7d** Q2 = 0.922; **Supplementary Figure S8b**, p<0.001, FDR<0.001).

**Figure 6:**
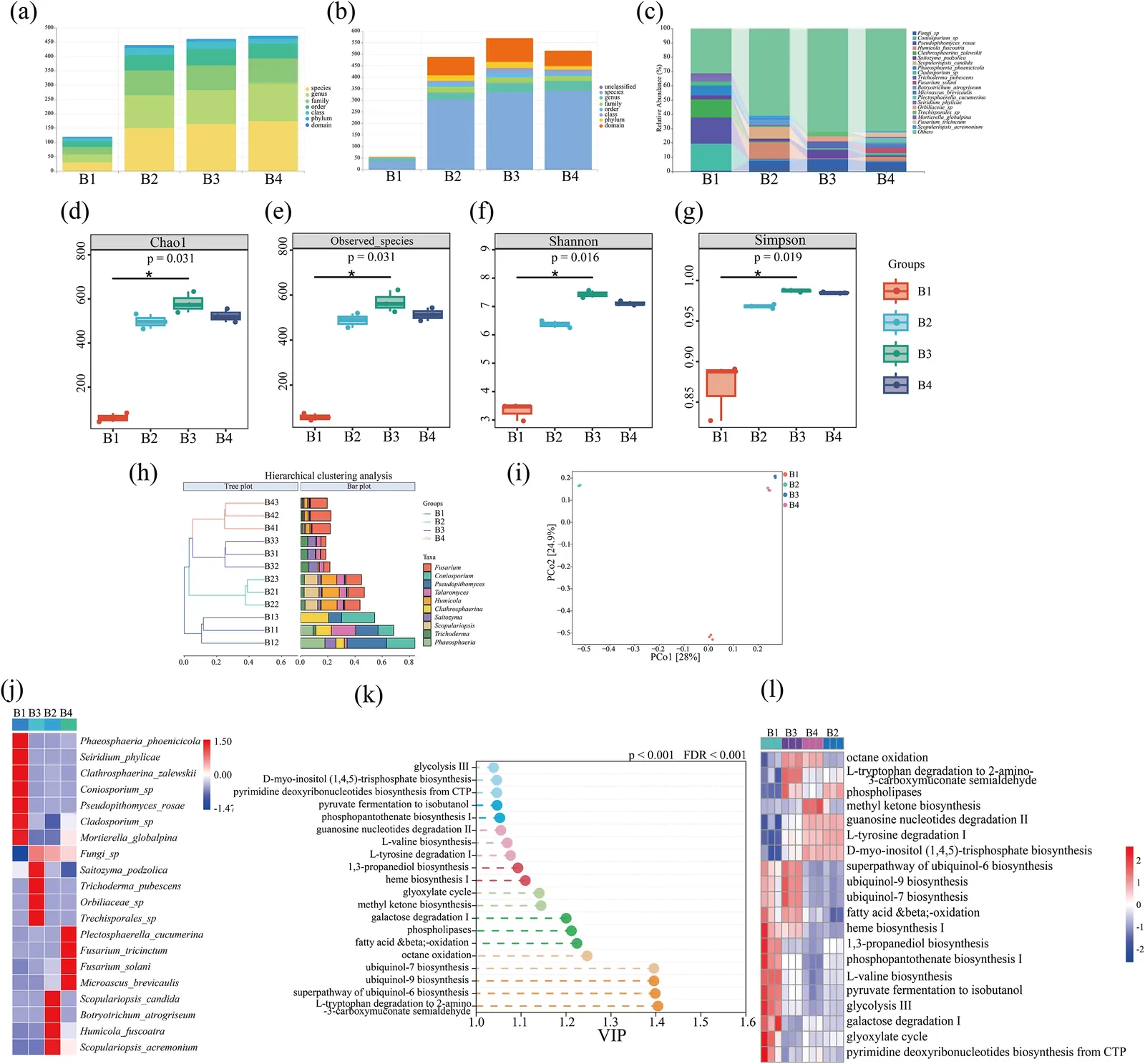
Fungal community composition and functional analysis. **(a)** Quantitative statistics of fungal taxonomic units. **(b)** Statistical overview of fungal species taxonomic annotations. **(c)** Statistical analysis of fungal community composition at the species level. **(d–g)** Analysis of α-diversity in fungal communities, using Chao1 and Observed species indices to evaluate richness, and Shannon and Simpson indices to evaluate diversity. **(h)** β-diversity (between-community diversity) clustering analysis of fungal communities. **(i)** PCoA classification of fungal community β-diversity, with a 95% confidence interval. **(j)** Heatmap depicting differential fungal community composition at the genus level, with red indicating high abundance and blue indicating low abundance. **(k)** Prediction and top 20 selection of significantly differential metabolic pathways in fungal communities, with statistical significance at p<0.001. **(l)** Heatmap display of the top 20 significantly differential metabolic pathways in fungal communities, indicating high expression with red and low expression with blue.

In summary, the rhizosphere soil microbial community structures of B3 and B4 are more similar, which may be one of the reasons for their more similar secondary metabolic patterns. The rhizosphere fungal community compositions among the four *Atractylodes macrocephala* of quality grades showed more significant differences compared to bacterial communities, exhibiting stronger grades specificity. This suggests that structural variations and functional differentiation of fungal communities may have more critical impacts on the regulation of secondary metabolic pathways in *Atractylodes macrocephala*.

### Mantel test and spearman correlation analysis between soil environmental factors and secondary metabolic pathways

To investigate the specific driving mechanisms of soil environmental factors on differences in secondary metabolic pathways in *Atractylodes macrocephala*, we selected six significantly different metabolic pathways as primary research targets. For analytical purposes, we selected the top ten contributing secondary metabolites as representatives to conduct spearman correlation analyses with soil physicochemical properties, enzyme activities, and microbial community functional potential, respectively (**Figure 7a**). The results revealed that the secondary metabolites of *Atractylodes macrocephala* were closely associated with soil environmental factors: secondary metabolic factors (Formononetin, Xanthosine, Phloretin, Aesculetin, Rotenone) and signal transduction metabolic factors (Jasmonic acid, L-histidine, Guanosine) exhibited significant “mirror” distribution patterns. Notably, soil physicochemical properties (pH, NO^-3^-N, TN) and enzyme activities (S-SC, S-UE) showed significantly positive correlations with secondary metabolic factors (p < 0.05) and significantly negative correlations with signal transduction metabolic factors (p < 0.05), while microbial community diversity exhibited consistent trends (p < 0.05). Regarding microbial functional composition, seven fungal functions and nine bacterial functions demonstrated identical correlation patterns, whereas the remaining microbial functions showed opposite trends. Specifically, bacterial galactose metabolism and fungal 1,3-propanediol biosynthesis were prominently negatively correlated with secondary metabolic factors (p < 0.05) but positively correlated with signal transduction metabolic factors (p < 0.05). Additionally, Spearman analysis revealed significant interactions among soil environmental factors, indicating that microbial community composition and functional potential were significantly correlated with soil physicochemical properties and enzyme activities (**Figure 7b**). Since spearman analysis had verified significant correlations among individual factors, we further employed mantel tests to analyze the overall effects of all environmental factors on the 6 metabolic pathways (**Figures 7c–e**). The results demonstrated that soil physicochemical factors and enzyme activities—particularly S-UE, NH_4_^+^-N, NO^-3^-N, TN, and pH—exerted extremely significant effects on these 6 metabolic pathways (p < 0.01). Microbial community structure and function also exhibited extremely significant impacts, with fungal communities showing more significant correlations with the 6 metabolic pathways (p < 0.05, r > 0.2) compared to bacterial communities, suggesting that fungi exerted stronger influences on *Atractylodes macrocephala* metabolic pathways than bacteria. RDA results further confirmed these findings (**Supplementary Figures S9a, b**, p < 0.01). In conclusion, soil nitrogen source signaling, pH, along with microbial community structure and function emerged as key driving factors causing the differentiation of secondary metabolic pathways in *Atractylodes macrocephala*

**Figure 7:**
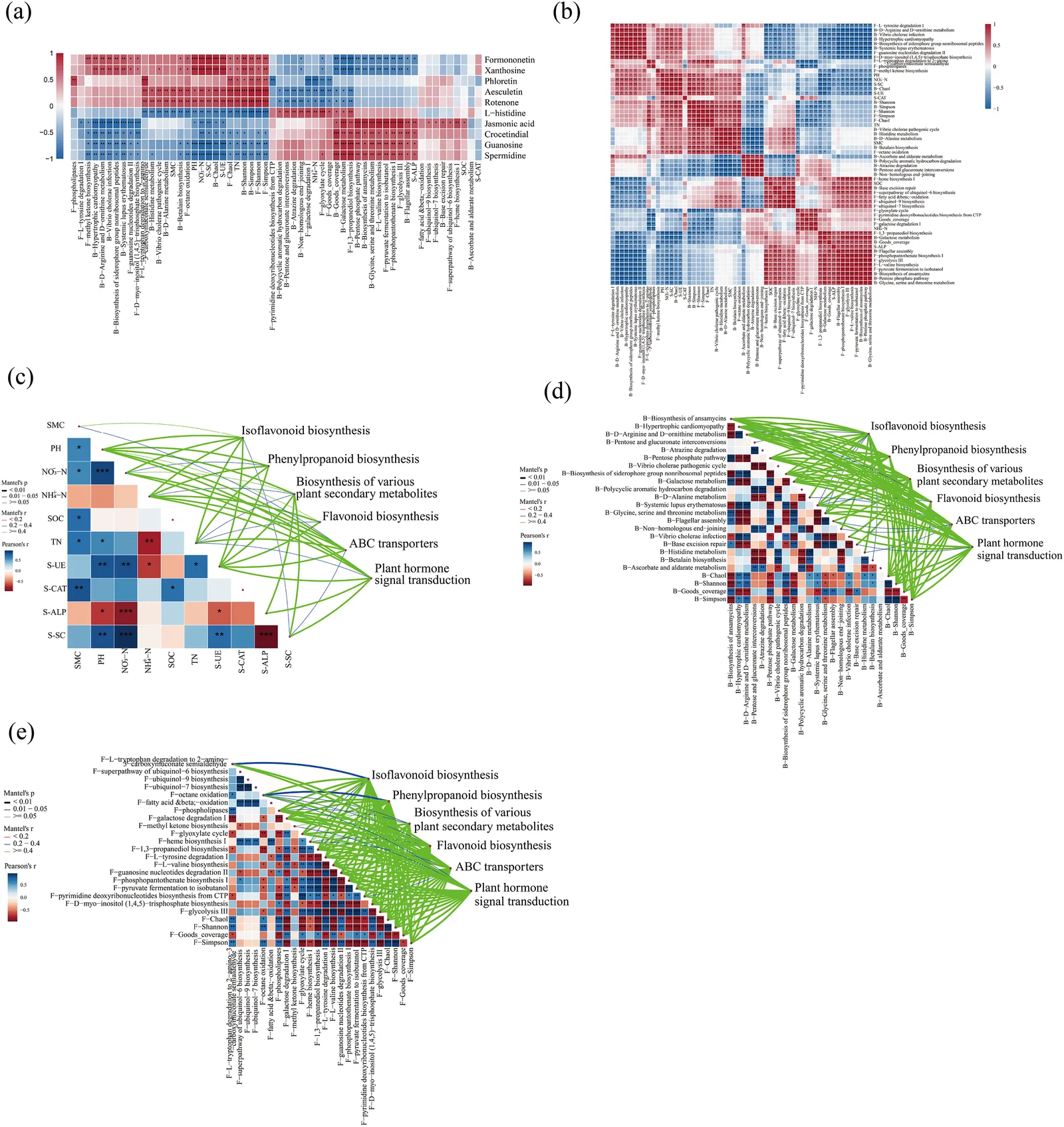
Correlation analysis and mantel correlation test between rhizosphere soil environmental factors and secondary metabolic pathways in *Atractylodes macrocephala*. **(a)** Spearman rank correlation analysis between soil environmental factors and representative significantly differential secondary metabolites, with “*” = p<0.05, “**” = p<0.01, and “***” = p<0.001. **(b)** Spearman rank correlation analysis among various soil environmental factors. **(c–e)** mantel test illustrating the relationship between soil physicochemical properties and enzyme activities, bacterial community diversity and function, fungal community diversity and function, and the significantly differential secondary metabolic pathways in Atractylodes macrocephala.

## Discussion

### Exploration of differential response mechanisms in the secondary metabolic pathways of *Atractylodes macrocephala*

As a major medicinal material, the efficacy quality of *Atractylodes macrocephala* is primarily determined by the accumulation of its secondary metabolites. This study demonstrated that significant differences exist in the secondary metabolic pathways and predominant secondary metabolites of *Atractylodes macrocephala* across different cultivation age, with these differences being predominantly regulated by phytohormone signaling and transport pathways. These processes activate the phenylpropanoid, flavonoid, and isoflavonoid biosynthesis pathways, consequently exerting cascade effects on the expression of other downstream secondary metabolites. Secondary metabolism represents a core physiological mechanism by which medicinal plants adapt to environmental stresses, with its metabolites being the products of long-term interactions between plants and their environment (Li, 2012). Based on statistical analysis revealed correlations between soil environmental factors and secondary metabolism data, enabling us to develop a model for the interaction between *Atractylodes macrocephala* and rhizosphere soil: the rhizosphere soil environment interacts extensively with the plant, and the evolution of this environment induces hormone signaling responses dominated by JA during *Atractylodes macrocephala* cultivation. This signal is mediated through ABC transport pathways, activating major defense metabolic pathways including phenylpropanoid, isoflavonoid, and flavonoid biosynthesis, thereby indirectly regulating other metabolic pathways such as sesquiterpenoid biosynthesis. Concurrently, certain synthesized secondary metabolites are secreted back into the rhizosphere through root exudates, reshaping the microbial community structure and modulating soil nutrient conditions, thus forming a feedback loop between plant metabolism and the rhizosphere environment (**Figure 8**). Additionally, the main medicinal components of *Atractylodes macrocephala*—Atr I, II, and III—belong to the sesquiterpenoid class and represent branches in the biosynthesis of various plant secondary metabolites. This also suggests that long-term monoculture may potentially affect the primary efficacy of *Atractylodes macrocephala*. Although different developmental stages can influence the secondary metabolism of *Atractylodes macrocephala*, including carbon allocation and root exudates like sugars and organic acids, which in turn affect rhizosphere microbial diversity (Bai et al., 2010; Li et al., 2025). However, this process is also influenced by soil environmental factors, and it is not driven by a single factor alone. Secondary metabolites are defensive compounds produced by plants in response to biotic and abiotic stresses, and their full activation often requires environmental signals as triggers or amplifiers. In this study, we collected *Atractylodes macrocephala* roots and rhizosphere soil in November during the mature stage, minimizing the potential interference caused by different developmental stages.

**Figure 8:**
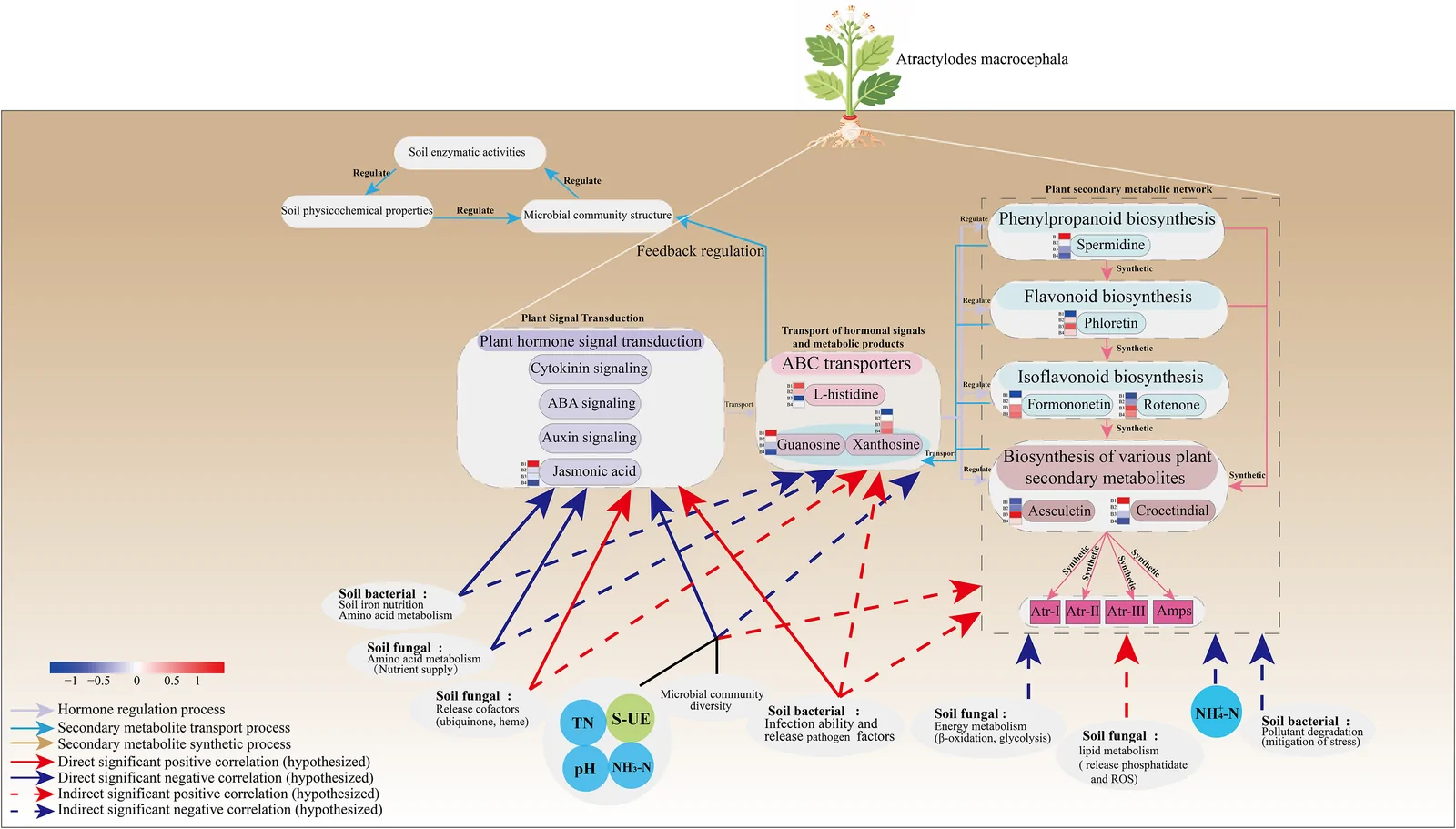
The synergistic regulatory mechanism of soil environmental factors on the secondary metabolism of *Atractylodes macrocephala*. The diagram illustrates how soil physicochemical properties and enzyme activities, along with the diversity and function of fungal and bacterial communities, regulate the significantly differential secondary metabolic pathways in *Atractylodes macrocephala*. The influence of rhizosphere soil environmental factors on the hormone pathway of *Atractylodes macrocephala* is direct, whereas their regulation of the ABC transport pathway and the phenylpropanoid biosynthesis pathway is indirect. Additionally, the secondary metabolites produced via the phenylpropanoid biosynthesis pathway are transported by the ABC transport pathway through root exudates, impacting the composition of the rhizosphere soil microbial community and indirectly adjusting soil nutrient status.

### Analysis of epigenetic variation in *Atractylodes macrocephala* of different quality grades

Epigenetic variation serves as a critical mechanism for medicinal plants to adapt to environmental conditions, with its diversity directly reflecting the epigenetic structural stability and risk resistance capacity of populations in complex habitats (Han et al., 2007). This study revealed that four *Atractylodes macrocephala* of quality grades maintained high epigenetic diversity, indicating stable epigenetic structures and strong viability without evident bottleneck effects. This suggests that seed preservation propagation can effectively conserve desirable traits and continuously yield high-quality *Atractylodes macrocephala* medicinal materials. Further analysis demonstrated that *Atractylodes macrocephala* exhibited abundant epigenetic diversity and significant differentiation at the epigenetic level. PCoA revealed reduced sample overlap at the epigenetic level, exhibiting distinct grades specificity. This provides strong evidence that *Atractylodes macrocephala* of different quality grades exhibit epigenetic variations, with particularly notable differences in DNA methylation patterns. Conversely, epigenetic regulation characterized by rapid response capabilities acts as a crucial mechanism for plants to respond to habitat stresses (Liu and Able, 2022; Turgut et al., 2020). Our findings confirm that *Atractylodes macrocephala* can adjust its DNA methylation in response to soil environmental changes, potentially influencing its growth and development.

### Synergistic regulatory mechanism of soil physicochemical properties and enzyme activities on the secondary metabolism of *Atractylodes macrocephala*

This study reveals through correlation analysis between datasets that soil nitrogen nutrition and pH value are key environmental factors affecting the secondary metabolic pathways of *Atractylodes macrocephala*, with the regulatory effect of nitrogen nutrition being particularly significant. Based on the carbon-nitrogen balance hypothesis, soil nitrogen levels modulate the plant’s carbon source allocation strategies: under nitrogen-sufficient conditions, plants prioritize supporting growth and development while inhibiting defensive secondary metabolic processes; whereas under nitrogen-deficient conditions, they accumulate relevant secondary metabolites through induced defense mechanisms (Li et al., 2026). The results from mantel tests and spearman correlation analyses support this mechanism, showing a significant negative correlation between NH_4_^+^-N content and the defensive secondary metabolic pathways of *Atractylodes macrocephala* (**Figure 8**, p < 0.05). From the perspective of soil nitrogen transformation processes, TN content positively correlates with urease activity, with S-UE decomposing urea to produce NH_4_^+^-N, which subsequently undergoes nitrification to generate NO_3_^--^N—the two primary nitrogen sources for plants. However, NH_4_^+^-N accumulation feedback-inhibits S-UE activity. Therefore, when nitrogen transformation proceeds smoothly, it often exhibits high S-UE activity accompanied by high NO_3_^--^N accumulation, while NH_4_^+^-N content remains relatively low, explaining the opposite correlation trends observed between S-UE, NO_3_^--^N, TN, and NH_4_^+^-N ((**Figure 8**). At the plant physiological level, higher soil nitrogen availability (high S-UE, NO_3_^--^N, and TN) tends to inhibit the induction of defensive hormones (such as JA), thereby indirectly promoting the synthesis and translocation of auxins to prioritize plant growth and development. This process, while inhibiting the phenylpropanoid metabolic pathway, activates the flavonoid/isoflavonoid biosynthetic pathway, whose products maintain normal plant growth and cellular homeostasis by feedback-inhibiting auxin overaccumulation and scavenging reactive oxygen species (Xu et al., 2024). This metabolic regulatory difference based on nitrogen form variations is another important reason why related indicators exhibit opposite correlations with NH_4_^+^-N. Continuous monoculture often leads to soil acidification, hindering plant growth and component accumulation, and is one of the primary causes of continuous cropping obstacles (Xu et al., 2024). In this study, the soil pH values of all samples remained above 5.0, indicating that current cultivation practices effectively suppress soil acidification. Further mechanistic analysis revealed that pH values positively correlated significantly with the secondary metabolic pathways of *Atractylodes macrocephala* (p < 0.05) and negatively correlated significantly with signaling/transduction pathways (p < 0.05), suggesting that acidic environments may inhibit the synthesis of major secondary metabolites in *Atractylodes macrocephala* while simultaneously inducing the production and transmission of defense signals through metal ion stresses such as aluminum ions.

### Impact mechanism of soil fungal community structure and function on the secondary metabolism of *Atractylodes macrocephala*

Rhizosphere microbiota, as critical components of soil systems, profoundly influence plant secondary metabolism by driving nutrient transformation and cycling (Yuan et al., 2019). Previous research has confirmed that compared to bacteria, the structure and function of rhizosphere soil fungal communities in *Atractylodes macrocephala* have a decisive impact on its secondary metabolic pathways, with the role of community diversity being particularly significant. Based on functional prediction and mantel test, we propose that soil fungi reshape rhizosphere microbial carbon and nitrogen cycles through energy metabolism (β-oxidation, glycolysis, glyoxylate cycle), cofactors (ubiquinone, heme), as well as amino acid and lipid metabolism (nutrient supply, release of phosphatidic acid and Reactive Oxygen Species (ROS)), thereby stimulating plant hormone signal transduction (**Figure 8**).The regulatory effects of fungal community function on *Atractylodes macrocephala* secondary metabolism are primarily manifested in two dimensions: “resource competition” and “defense induction”. Regarding resource competition and nutrient utilization, fungal energy metabolism exhibits a significant indirect negative correlation with *Atractylodes macrocephala* secondary metabolism (p < 0.05). This is because high-intensity fungal energy metabolism competes with plants for substrates such as fatty acids and glucose. Under long-term interactions, plants inhibit this process through root exudates to ensure their own resource acquisition, while high energy metabolism in fungal communities generally indicates a collapse of plant defense capabilities. Similarly, fungal amino acid metabolism supplements soil nutrients, prompting plants to prioritize resource allocation toward growth and development rather than defense, thus showing a significant negative correlation with signaling/transduction pathways (p < 0.05). In terms of defense induction, fungal cofactor synthesis demonstrates a direct significant positive correlation with plant signaling/transduction pathways (p < 0.05). Enhanced cofactor synthesis reflects increased fungal infection potential, thereby eliciting strong defensive signals in plants. Furthermore, fungal lipid metabolism generates virulence factors such as phosphatidic acid and ROS, significantly enhancing induced defense responses in plants, which indirectly promotes the synthesis of secondary metabolites.

### Impact mechanism of soil bacterial community structure and function on the secondary metabolism of *Atractylodes macrocephala*

Although bacterial community diversity significantly influences the secondary metabolic pathways of *Atractylodes macrocephala*, the overall effect of soil bacterial communities and their functions is relatively weak compared to fungi, with more factors showing no significant correlation (p > 0.05) with the major differential metabolic pathways. Functional predictions and mantel test outcomes revealed that rhizosphere bacteria primarily regulate secondary metabolism through two pathways: “environmental improvement” and “biotic stress” (**Figure 8**). In terms of environmental improvement, bacteria ameliorate soil iron nutrition through siderophore secretion, thereby alleviating iron deficiency stress and reducing the production and translocation of defense hormones, a finding that has been validated in previous studies (Trapet et al., 2016). Concurrently, their amino acid metabolism and pollution degradation functions optimize the rhizosphere nutrient environment and reduce toxicity, prompting plants to allocate more resources to growth and development rather than defense, resulting in significant negative correlations (p < 0.05) between these factors and defense signaling/transduction as well as secondary metabolism. Conversely, in the context of biotic stress, the infectivity and virulence factor release of bacteria act as exogenous stimuli, rapidly activating plant immune responses, inducing JA signaling, and consequently enhancing defensive metabolism, which shows significant positive correlations (p < 0.05) with related metabolic pathways. Additionally, higher diversity in soil microbial communities typically indicates smooth soil nutrient cycling and adequate nutrient availability, leading to *Atractylodes macrocephala* allocating more resources to growth and development. This also necessitates the production of metabolites such as flavonoids to suppress auxin accumulation, thereby exhibiting negative correlations with defense mechanisms. The spearman correlation analysis results have confirmed these relationships.

## Conclusion

This study demonstrates that *Atractylodes macrocephala* can adapt to varying soil environments by modulating its DNA methylation patterns, while maintaining a high degree of epigenetic diversity. Soil nitrogen nutrition and pH value emerge as critical environmental driving factors that most strongly associated with metabolic pathways through carbon-nitrogen balance and hormonal signaling (such as JA). Efficient nitrogen transformation and weakly acidic environmental conditions are more conducive to the accumulation of secondary metabolites. Rhizosphere fungi, as core microbial drivers, modulate the secondary metabolic pathways of *Atractylodes macrocephala* through “resource competition” and “defense induction,” whereas bacteria primarily play auxiliary roles in “environmental amelioration” and “biotic stress.” Collectively, the synthesis and accumulation of secondary metabolites in *Atractylodes macrocephala* are under the coordinated regulation of a multi-dimensional network encompassing “soil environment-rhizosphere microbiota”. Nevertheless, this study has several limitations. Future research should conduct more comprehensive analyses and validations of soil environmental factors regulating the metabolic pathways of *Atractylodes macrocephala*, while exploring the underlying micro-level regulatory mechanisms at the epigenetic level.

## Data Availability

The data provided in this study have been archived in the NCBI Sequence Read Archive repository, with accession numbers PRJNA1533652 and PRJNA1533677.
