# Supplementary material for: Research on the epigenetic alterations in Atractylodes macrocephala and the synergistic regulatory mechanism of “soil environment-rhizosphere microbes” on its secondary metabolism
Source: Front Plant Sci. 2026 Oct 1;17:1951327. doi: 10.3389/fpls.2026.1951327 (PMC13668753; doi:10.3389/fpls.2026.1951327)
Supplement: Supplementary file 1 [file SupplementaryFile1.docx]

**Fig.S1 Display of *A. Macrocephala* Samples with Varied Qualities**

**Fig.S2 Polyacrylamide Gel Electrophoresis Diagram of MSAP Primer Amplifications.** (a) E2-H7 Primer. (b) E3-H10 Primer. (c) E2-H4 Primer. (d) E4-H8 Primer.

 **Fig.S3 HPLC-MS Fingerprinting of Secondary Metabolites in *A. Macrocephala* with Different Qualities.**

**Fig.S4 RSD of QA Results.** In QC samples, approximately 65% of the characteristic peaks have an RSD of less than 30%, indicating good data quality. The left vertical axis shows the proportion of peaks, the right vertical axis represents the number of peaks, and the horizontal axis indicates the range of RSD values.

**Fig.S5 Venn Diagram Illustrating the Secondary Metabolites in *A. Macrocephala* with Different Qualities.** The left bar graph shows the number of distinct metabolites identified in each comparison group. The dots and lines below the diagram represent the selected sets of comparison groups. The right bar graph displays the number of differential metabolites commonly identified exclusively within the selected sets of comparison groups.

**Fig.S6 Screening and Analysis of Differential Secondary Metabolites.** (a)-(b) PCA for Classification Based on the Relative Abundances of Secondary Metabolites. (c)-(d) In the PLS-DA Permutation Test Plot, the reliability and validity of the results are indicated if either of the following conditions is met: 1. All blue Q2 points are lower than the rightmost original blue Q2 point from left to right (noting that the rightmost blue Q2 point may overlap with the green R2 point in the upper right corner); 2. The regression line of the points intersects the vertical axis or is below.

Fig.S7 **Analysis of Differences in Microbial Communities and Screening for Differential Metabolic Pathways.** (a) LEfSe for Identifying Differential Bacterial Communities. (b) Permutation Test for Screening Bacterial Metabolic Pathways with Significant Differences. (c) LEfSe Analysis for Identifying Differential Fungal Communities. (d) Permutation Test for Screening Fungal Metabolic Pathways with Significant Differences.

**Fig.S8 Functional Prediction of Microbial Communities.** (a) Functional Prediction for Bacterial Communities. (b) Functional Prediction for Fungal Communities.

**Fig.S9 RDA Analysis Between Soil Environmental Factors and Different Metabolic Pathways of *A. Macrocephala*.** (a) Redundancy analysis between soil physical and chemical properties and enzyme activities and different metabolic pathways of *A. Macrocephala*. (b) Redundancy analysis between soil bacterial functions and different metabolic pathways of *A. Macrocephala*. (c) Redundancy analysis between soil fungal functions and different metabolic pathways of *A. Macrocephala*.

Table S1 Primer Sequences for MSAP

primer	sequence (5’-3’)

*EcoR* I adaptor top	CTCGTAGACTGCGTACC

Single stranded

*EcoR* I adaptor bottom	AATTGGTACGCAGTC

*Hpa* II/*Msp* I adaptor top	GATCATGAGTCCTGCT

adapter

*Hpa* II/*Msp* I adaptor bottom	CGAGCAGGACTCATGA

E0	GACTGCGTACCAATTCA

Pre-amplification

HM0	ATCATGAGTCCTGCTCGG

E2	GACTGCGTACCAATTCAAG

E3	GACTGCGTACCAATTCACA

E4	GACTGCGTACCAATTCACT

Selective screening

HM2	ATCATGAGTCCTGCTCGGTCG

HM4	ATCATGAGTCCTGCTCGGTTC

Amplification

HM7	ATCATGAGTCCTGCTCGGTGA

HM8	ATCATGAGTCCTGCTCGGTGT

HM9	ATCATGAGTCCTGCTCGGTGC

HM10	ATCATGAGTCCTGCTCGGTAC

Table S2 DNA Methylation Profiles in Various Populations of *A.Macrocephala*

| Population | Band count Methylation type |
| --- | --- |
|  | Ⅲ type Ⅱ type Ⅰ type Full (%) Hemi (%) Toal (%) |
| B1 354 525 529 25.14 37.29 62.43  B2 402 423 747 25.57 26.91 52.48  B3 418 410 602 29.23 28.67 57.90  B4 414 518 685 25.60 32.03 57.64 | |

Full(%): Fully Methylated regions; Hemi: Hemi Methylated regions; Toal: Global Methylation.
